# Reactive Sputtering of Aluminum Nitride (002) Thin Films for Piezoelectric Applications: A Review

**DOI:** 10.3390/s18061797

**Published:** 2018-06-02

**Authors:** Abid Iqbal, Faisal Mohd-Yasin

**Affiliations:** Queensland Micro- and Nanotechnology Centre, Griffith University, Nathan, QLD 4111, Australia; abid.iqbal@griffithuni.edu.au

**Keywords:** physical vapor deposition, sputtering, aluminum nitride, piezoelectric, energy harvester

## Abstract

We summarize the recipes and describe the role of sputtering parameters in producing highly c-axis Aluminum Nitride (AlN) films for piezoelectric applications. The information is collated from the analysis of around 80 journal articles that sputtered this film on variety of substrate materials, processes and equipment. This review will be a good starting point to catch up with the state-of-the-arts research on the reactive sputtering of AlN (002) thin film, as well as its evolving list of piezoelectric applications such as energy harvesters.

## 1. Introduction

Energy harvesting or energy scavenging is the process of extracting energy from the ambient sources in the environment. Energy harvester, instead of battery usage, is preferred for powering wireless sensors because the latter is limited by its limited life span. The ambient sources are broadly divided into four categories: Solar, thermal, wind, and mechanical vibration [1]. Researchers have investigated different methods to convert them into electrical energy via resonant and non-resonant devices [2]. A variety of transduction schemes have been proposed using solar, thermoelectric, electromagnetic, piezoelectric, capacitive etc. Among them, piezoelectric emerges as one of the most practical solutions. It does not require external power sources for polarization in comparison to the electrostatic transductions. Furthermore, the micro-scale fabrication processes are simpler compared to the electromagnetic transductions [3]. 

Piezoelectricity is derived from the Greek word “piezo”, which means “to squeeze”. It is the property of the crystalline materials that develop an electric dipole when the mechanical strain is applied to them. Conversely, they exhibit an induced mechanical strain when being subjected to an electric potential. In dielectrics, the electrons are strongly tied to the outermost atomic shells and form a symmetrical cloud around the nucleus in the absence of an electric field. In the non-polar dielectric, the electric field polarizes the atom or the molecules in the material by moving the center of the electron cloud away from the nucleus, resulting in the formation of an electric dipole. All of these individual dipoles add up over the entire crystal and produce the net polarization that results in electric field generation across the material [4]. 

**Figure 1:**
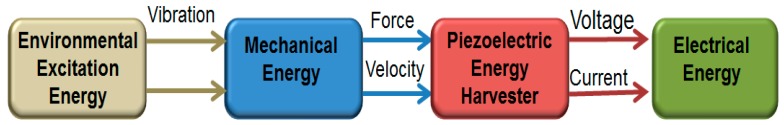
Piezoelectric transduction of mechanical to electrical energies.

Figure 1 shows the block diagram to convert the mechanical to the electrical energies using piezoelectric transduction. There are three major steps. First, the mechanical energy is absorbed from the ambient environment. It can be in the form of vibration, force, or motion. Then, this energy is piezo-electrically transduced to electrical voltage and current. Finally, the electrical energy is rectified and stored [5]. 

Piezoelectric materials are classified as single crystal, polycrystalline, polymers, ceramics, and thin films. The selection of the most suitable forms depends on the availability of the deposition methods, the process complexities, and their compatibility with the targeted applications. Thin films have a substantial range of advantages such as low hysteresis, high sensitivity, low power requirements, and the ability to generate large mechanical deflections. The three most commonly used piezoelectric thin films are lead zirconate titanate (PZT), zinc oxide (ZnO), and aluminum nitride (AlN).

PZT film is preferred in piezoelectric applications due to its high electromechanical coupling coefficient (*k*^2^) and piezoelectric coefficient (d_ij_). However, the presence of lead in this material results in higher probability of contamination in the clean room during its processing. ZnO and AlN possess similar values of *k*^2^ and d_ij_. However, the performance of ZnO degrades significantly at high temperature due to its low Curie point. As a result, AlN trumps ZnO in the fabrication of the micro-scale piezoelectric devices at such temperature.

AlN thin films can be produced via various methods including metal organic chemical vapor deposition (MOCVD), molecular beam epitaxy (MBE), electron cyclotron resonance dual-ion beam sputtering, and pulsed laser ablation. However, these methods are expensive and the high processing temperatures are required. Direct current (DC) and radio frequency (RF) sputtering have the advantages of being low temperature and low-cost, making them suitable for the fabrication of Micro-Electro-Mechanical Systems (MEMS) devices. 

Amongst MEMS applications, many groups are looking at AlN thin films to make piezoelectric energy harvesters. This development makes the review of the latest published works on the sputtering of AlN thin film timely. The information has been collated from around 80 articles that were published in well-known thin film journals. We would like to acknowledge that Iriarte et al. wrote a review article that detailed the sputtering parameters that influence the formation of c-axis AlN film in 2010 [6]. In order to produce definitive trends, they used pulsed DC sputtering to deposit AlN films on a variety of substrates such as Si, quartz, AlO_2_, MgO, MgAl_2_O_4_, and diamond. They did not however compile a list of sputtering recipes from other researchers. Therefore, we will provide those information. We would also like to state that this review only covers reactive sputtering of hexagonal AlN structures. Even though there are several works on the sputtering of cubic AlNs, we do not cover them here. 

The rest of the paper is as follows. Section 2 describes the AlN crystal structure. Then, Section 3 lists some of the structural, optical, thermal, and piezoelectric properties of AlN. After that, Section 4 describes the basic sputtering process for AlN film for the benefit of the non-experts. Advanced users should skip this section. Section 5 is the “meat” of this review. It contains the table that summarizes all the recipes from the journal articles. The rest of that section describes the roles of sputtering parameters towards producing highly c-axis AlN films. Several articles also discussed the piezoelectric applications, for examples surface acoustic wave devices [7], bulk acoustic wave devices [8], and energy harvesters [9]. Section 6 concludes by emphasizing the inter-dependency of those parameters towards depositing the highly c-axis films.

## 2. AlN Crystal Structure

**Figure 2:**
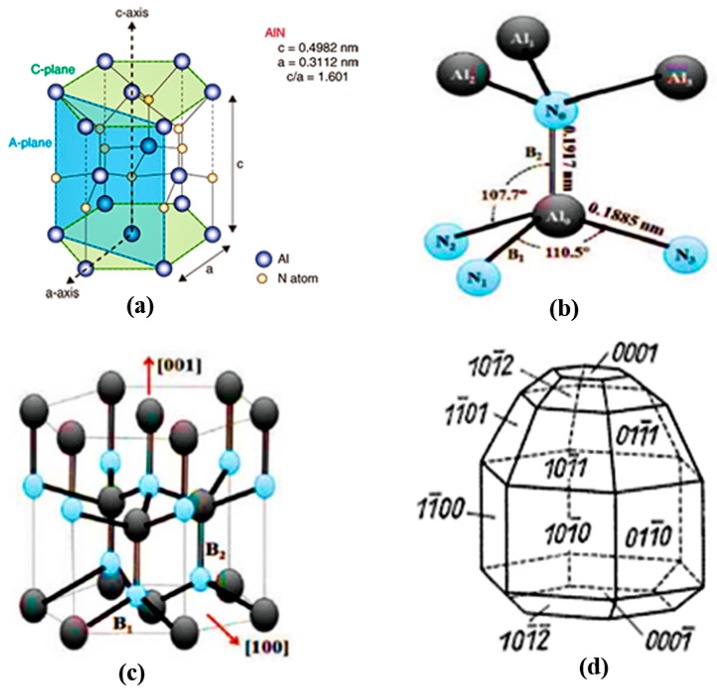
(**a**) Crystal structure; (**b**) B1 and B2 bonds; (**c**) crystal structure with B1 and B2 bonds; and (**d**) different planes of AlN.

**Figure 3:**
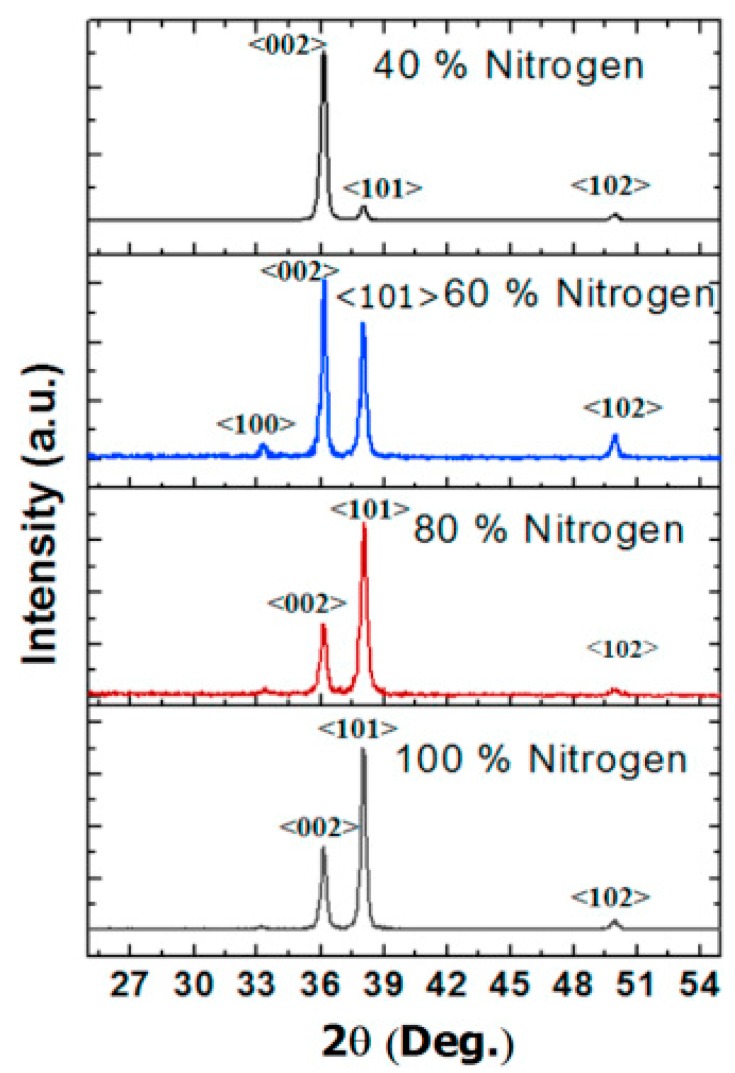
The example of XRD plot of AlN films [8]. There are four panels in the figure, denoting the crystal orientations of four films that were deposited with different Nitrogen concentrations. The AlN peaks are (100), (101), (102) and (002). The y-axis has an arbitrary unit.

AlN belongs to group III–V semiconductor family because it has a hexagonal closed-packed wurtzite structure [10]. The lattice parameters range from 3.110 to 3.113 Å for the a-axis, and from 4.978 to 4.982 Å for the c-axis. The c/a ratio varies between 1.600 and 1.602. This deviation from the ideal wurtzite structure is credited to its lattice instability [11]. Figure 2 shows its crystalline structure, bonds configuration, and different planes. Each Al atom is surrounded by four N atoms to form a tetrahedron with three B_1_ bonds between Al-N_(i=1,2,3)_ and one B_2_ bond between Al-N_0_ bond. The bond lengths of B_1_ and B_2_ are 0.1885 and 0.1917 nm, respectively. The bond angles for N_0_-Al-N_1_ and N_1_-Al-N_2_ are 107.7° and 110.5°, respectively. The (100) plane is composed of the B1 bonds, while the (002) and (101) planes consist of the B1 and B2 bonds [12]. Figure 3 shows the example of XRD plot of AlN films. This is taken from one of authors’ own article [8]. The 2θ peak positions for the (100), (101), (102), and (002) planes are clearly labeled. For example, the peak for (002) plane is at 2θ of 36°.

## 3. AlN Properties 

Some of the structural, optical, thermal, and piezoelectric properties of bulk AlN are given in Table 1. These data are taken from references [13,14]. It should be clear that these data are not comprehensive, and there are a lot more mechanical and corrosion properties that we do not cover in Table 1. AlN has a large energy band-gap of 6.2 eV, a high thermal conductivity of 180 W/(m·°K), a high breakdown voltage of 1.2 × 10^6^ V/cm, and a high resistivity of 10^15^ Ohm·cm. It also has a high surface acoustic Wave (SAW) velocity of 12,000 m/s and a moderate *k*^2^ of ~1% [15]. The value of the latter parameter is highest along the (002) plane, prompting piezoelectric researchers to target for the production of a highly c-axis AlN film. Furthermore, AlN has higher values of mechanical, thermal and chemical stability compared to the other members of the III-V nitride semiconductors. Later in Section 5, we will highlight few works that sputtered AlN films and characterized their performances at those conditions.

## 4. Overview of Sputtering Process

**Figure 4:**
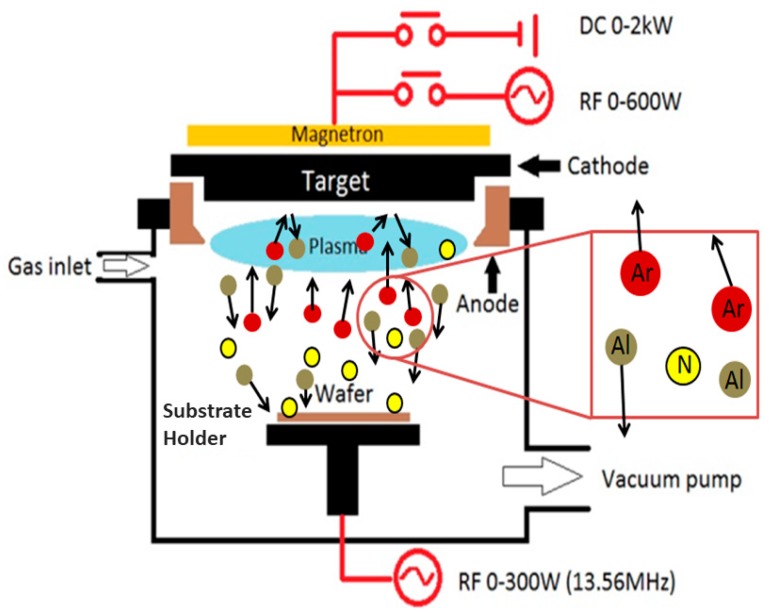
Reactive sputtering process for AlN film [16].

The generic sputtering process for AlN film is shown in Figure 4. This section is specifically written for non-experts, so that they could follow the discussions on the sputtering parameters in Section 5. The system consists of a chamber, gas inlet, vacuum pump, and power supply. The chamber houses the sputtering target and its shutter at the top, and wafer holder (or sometimes referred to as susceptor) at the bottom. The inlet feeds Argon (Ar) and Nitrogen (N_2_) gasses. The pump maintains the high vacuum condition in the chamber. The power supply could be DC, RF, or both. It feeds high voltage to the magnetron system that transmits electromagnetic waves from the cathode to the anode ring. In that process, the gasses inside the chamber are transformed into their plasma state. Initially, Ar is fed into the chamber through the inlet and gets ionized. After that, N_2_ is fed, undergoing the same transformation. Based on selection of the power supply, three sputtering modes could be set, namely reactive DC, reactive pulsed DC, and reactive RF modes. Suppose that we use reactive DC mode. The positive and negative potentials are applied to the anode and cathode (target), respectively. Both gasses will have positive and negative ions floating in the chamber. The positive ions are accelerated to the target, which is an Aluminum disc. The forceful collision between the target and these ions ejected the Al atoms. Under the “Poison condition”, the Al and N ions merge to create an AlN compound. They eventually condense into a solid state and becomes an AlN thin film on top of the wafer. In some sputtering equipment, the susceptor is electrically biased to attract the ions towards it. A separate RF source is used for this purpose.

## 5. Compilation of Recipes and the Roles of the Sputtering Parameters

Table 2 lists the published articles that deposited c-axis AlN films on top of several substrates using a variety of sputtering equipment and processes. We would like to emphasize that there are many others that are not included. These works are selected due to the interesting natures of their experimental setups and/or results. Each row lists the details of one particular article. The columns contain the following parameters: Author (year of publication), substrate material, sputtering type, sputtering power, substrate temperature, sputtering pressure, base pressure, N_2_/Ar ratio, total gas volume, distance between target to substrate, full width at half maximum (FWHM), deposition rate, thickness, surface roughness, and notes. This last column describes specific objectives for that particular work. 

The crystal quality of the AlN film is normally represented by its FWHM value. One of the columns in the table contains this data. It should be clear that the FWHM values specifically refers to the (002) orientation. However, this review is not able to rank the crystal quality of the films that are reported in all articles using this parameter because some groups used FWHM of the rocking curve, while others used FWHM of the diffraction peak. This distinction is clearly noted in Table 2. There were also a few older papers that characterized crystal quality in term of peak intensity (in count per second). This data was not included since this unit is known to be arbitrary. Nevertheless, other qualities of thin films such as the deposition rate, film thickness, and surface roughness are provided.

**Figure 5:**
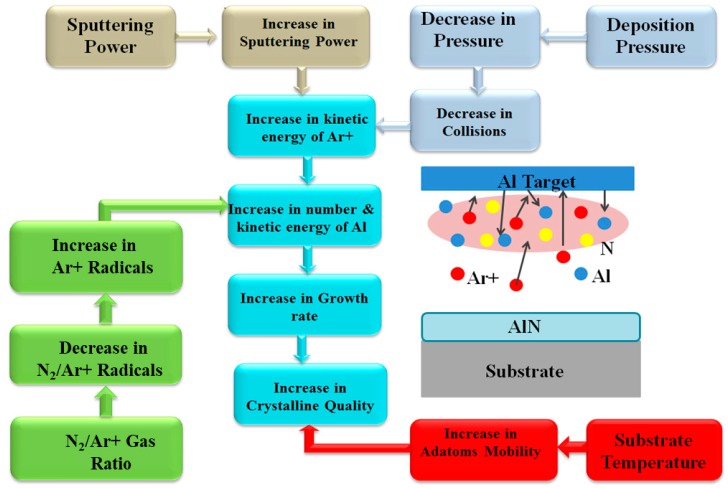
Flow chart on the role of sputtering parameters towards depositing c-axis AlN.

The data from Table 2 compiles the values of the sputtering parameters that had been used towards depositing highly c-axis AlN films. The reason for this compilation is as follows. These recipes could serve as reference points for any new work on the deposition of AlN film. Readers should be able to find the closest matches from Table 2 in term of their substrates and sputtering equipment. After locating the recipe(s), they should read the remaining sub-sections after Table 2 to understand the influences of different parameters to the deposition of highly c-axis film. The generic flow chart in Figure 5 summarizes them graphically, as well as highlighting the critical interactions between those sputtering parameters. With those information, reader should be able to commence the sputtering process, and subsequently alter their recipes based on the quality of the films.

The remainder of this section explains the roles of the sputtering parameters towards depositing the c-axis AlN films. The parameters are the choice of substrates, sputtering pressure, the sputtering power, the ratio of N_2_/Ar, the sputtering temperature, the film thickness, the distance between target to substrate, the substrate bias voltage, the base pressure, and the magnetic configuration. For each parameter, selected works that investigate its effect will be mentioned. In addition to using the works that were already summarized in Table 2, i.e., references [17,18,19,20,21,22,23,24,25,26,27,28,29,30,31,32,33,34,35,36,37,38,39,40,41,42,43,44,45,46,47,48,49,50,51,52,53,54,55,56,57,58,59,60,61,62], we also highlight additional articles that are not included in that table i.e., references [63,64,65,66,67,68,69,70,71,72,73,74,75,76,77,78,79,80,81,82,83,84,85,86] to illustrate the effect of specific parameters. 

Finally, we mention in Section 3 that AlN is a unique material in a sense that it has a high mechanical, thermal and chemical stability. Therefore, we will discuss a few works [87,88,89,90] in Section 5.11 that sputtered and characterized their AlN thin films for applications in difficult environments. This information will be helpful for the new research that would like to employ this material to make sensors/devices at those conditions.

### 5.1. Choice of Substrates

Numerous research groups have successfully deposited c-axis oriented AlN on a broad range of substrates such Si (100), Si (111), titanium, molybdenum, aluminium, c-sapphire, aluminium oxide, microcrystalline diamond, glass, silicon dioxide, copper, silicon carbide, and chromium, etc. Table 2 lists them as well as the sputtering parameters and the qualities of the sputtered AlN films. While Si remains the preferred choice, researchers opted for other materials chiefly to reduce the lattice mismatch and the coefficient of thermal expansion (CTE) between these substrates and the c-axis AlN film. For example, the authors of this review made our contributions by sputtering AlN film on top of Si substrates with cubic silicon carbide (3C-SiC) buffer layers [8,16].

This is such an important parameter, that the previous review paper on similar topic performed their own investigations on the successful deposition of c-axis AlN film using multiple substrates namely Si, quartz, AlO_2_, MgO, MgAl_2_O_4_, and diamond. Then, Iriarte et al. [6] compiled the empirical results from all samples to plot the trend of the rest of sputtering parameters towards depositing highly c-axis AlN film. 

### 5.2. Sputtering Pressure

According to the kinetic theory of molecular gasses, the ions, as well as the neutral species inside the sputtering chamber, have higher kinetic energies at lower process pressure. When they condense and are transformed to the solid state (called adatoms), they land on the surface of the substrate. The high kinetic energies create adatoms with faster mobility, which promote the high growth of c-axis AlN films [63]. It was also reported that when the sputtering pressure increased, the collision between sputtering particles and Ar ions led to the formation of AlN (100) films [64]. Both observations have been reported by many researchers in Table 2 such as in references [17,22,28,35,39,41,45].

One such works worth being mentioned in details herein. Cherng et al. [45] utilized a two-step deposition process to enhance the quality of deposited AlN on Si (100) using pulsed DC reactive sputtering. They used a smaller pressure of 0.8 mTorr for the initial nucleation for a period of 10 min. Afterwards, a second step was done at 2, 3.3, and 4.6 mTorr respectively. They observed that the two-steps sputtering resulted in smaller FWHM of the rocking curve as well as the smaller magnitude of residual stress. The value of the latter decreased from −926 to −317 MPa at a constant deposition rate of 36 nm/min. 

Another group figured out the exact sputtering pressure before the AlN film orientation switched from the (002) to (100) planes. Kar et al. [65] investigated the effect of sputtering pressure on the crystal orientation and the morphological properties of deposited AlN on top of a p-type Si substrate. They observed the improved crystal quality for the AlN (002) film from 1.5 to 4.5 mTorr. After 6 mTorr, the crystal orientation changed to the (100) plane. They also observed that the surface roughness increased from 1.56 nm to 3.24 nm with the increasing pressure. In addition, the grain size was 114 nm until 4.5 mTorr pressure, and then decreased to 80 nm at 6 mTorr. The same switching trend was observed by another group. Taurino et al. [42] switched the AlN film orientation from the (002) to the (101) plane by increasing the pressure from 3 to 18 mTorr.

This observation seems to be valid on a variety of substrate materials. Singh et al. [39] employed Glass, Si, oxidized Si, Al–SiO2–Si, Cr– SiO2–Si, and Au–Cr–SiO2–Si substrates. They varied the sputtering pressure from 5, 10, and 20 mTorr and concluded that low pressure was favorable for the highly crystalline c-axis oriented AlN. 

### 5.3. Sputtering Power

From experimental point of view, this is the easiest parameter to manipulate. Therefore, many research groups from Table 2 investigated the effect of this parameters towards producing highly c-axis AlN film [20,22,27,39,41,43,45,48,54,61]. A higher sputtering power means higher kinetic energies being supplied to the ions. In combination with the effect of sputtering pressure as explained in sub-section (5.2), many researchers employed high deposition power at low deposition pressure to get the best crystal-quality film. The power ranges from 100 W to 5.5 KW. However, several groups demonstrated that the high power can negatively affect the crystal quality of the AlN film because of an increase in the kinetic energies of the so-called secondary atoms. 

A sputtering power in the range of 300 W to 500 W is typically used in RF sputtering, while a power in the range of 1000 W to 1800 W is typically employed in D.C. sputtering. The sputtering power also depends on the substrate to target distance and the type of sputtering system. For the former, a lower power is needed for a shorter distance. 

In addition to the relationship between the sputtering power and crystal quality, Guo et al. [54] investigated the effect of RF power on the deposition rate, surface roughness, and optical transmittance. The last parameter refers to the fraction of incident light that passes via the AlN films. They observed that the deposition rate and the surface roughness increased and decreased, respectively, with the increase in RF power. The film exhibited around 75% optical transmission in the visible and ultraviolet ranges. From this optical data, they theorized that the increased RF power introduced defects in their film. 

Another group documented the effect of the sputtering power on the grain size and residual stress of the film. Kusaka et al. [66] employed D.C. magnetron sputtering to deposit AlN films on a glass substrate at various sputtering powers. They observed that the grain size and the crystal quality improved with increasing power. Further, they noted that tensile stresses were obtained at lower power, while large compressive stresses were achieved at higher power. 

Kumada et al. [67] documented the combined effect of sputtering power and N_2_ concentration in their studies. The former was varied from 200 to 900 W, and the latter from 30% to 70%. They produced AlN (101) and (002) poly crystals between 200–600 W at 50% N_2_ concentration, and AlN (002) film starting from 700 W power. 

Finally, it should be mentioned that a two-steps deposition technique was also employed by few researchers. Lin et al. [68] tried to obtain a highly c-axis oriented AlN thin film on top of 3C-SiC/Si (100) substrate. First, they deposited a 50 nm nucleation layer at pure nitrogen using an AC power of 3 kW. Then, they ramped up the power to 5.5 kW. They obtained AlN (002) film with the lowest FWHM of rocking curve values of 1.73°. 

### 5.4. N_2_ to Ar Ratio 

Before covering the previous works that varied this parameter, we would like to explain the basic reactive sputtering. It has three modes, which is determined by the N_2_/Ar ratio. At lower N_2_ concentration i.e., “metal mode”, Al target is marginally covered with nitride. It is characterized by high deposition rate and high cathode voltage. Once N_2_ concentration increases, the “transition mode” occurs. The target surface becomes partially reactive with N_2_, resulting in a slight decrease in the deposition rate. The cathode voltage starts to decrease steeply because the higher secondary electron emission of the nitride being formed at the cathode surface pulled down the plasma impedance. Finally, the “Poison mode” is achieved when the entire target surface is covered with AlN compound. The cathode voltage and deposition rate are at their minimum level, and remains constant even at increasing N_2_ concentration. One of our published articles characterized this trend clearly in terms of discharged voltage and current. Readers are referred to Figure 1 from reference [8]. 

Table 2 listed the N_2_ to Ar ratio for all listed works. Some groups used lower and some groups used higher N_2_ concentrations to deposit highly c-axis AlN film. The past review paper by Iriarte et al. [6] had the same observation. In general, there are two opposite theories to explain the need for higher or lower N_2_/Ar ratio to grow AlN (002) film. While both agree that the kinetic energies of the smaller-mass N_2_ particles and bigger-mass Ar particles played major role in transferring the adatoms to the surface of the substrate, they differed on the end results. The groups who propagated the use of lower N_2_ concentrations argued that the AlN atomic bondings, i.e., B1 and B2, could only be created when Ar particles with the higher kinetic energies dominate. The group who needed the pure N_2_ concentration argued that the closed-pack (002) plane could only be assembled with lower surface energies, where N_2_ particles dominate. The details of both theories and their implications could be read in reference [69]. It should be noted that this is our own paper. 

In this review paper, both factions are treated equally. First, we highlight the works from the groups that needed a lower N_2_/Ar ratio. Liu et al. [70] studied the effect of N_2_ concentrations in the range of 20% to 80%. They reported an FWHM of rocking curve value of 3.1° at 20% N_2_/Ar ratio. The value increase dramatically to 7.41° at 80% ratio. Zhong et al. [71] also reported the strong influence of the N_2_ concentration on the AlN films. They reported a decrease in the FWHM of the (002) diffraction peak amplitude when the N_2_ concentration increased from 25 to 75%. That effect is prominently displayed in Figure 1 of their article, Clement et al. [72] concurred and also observed the decreasing deposition rate at increasing N_2_ concentration. 

We would also highlight the groups that could only achieved the c-axis AlN film at higher N_2_ concentration. Kar et al. [52] and Cheng et al. [64] specifically studied the effect of N_2_ concentrations on AlN film on top of Si (100) substrates. Both reported that at low N_2_ concentration, a strong (100) peak was observed. The increase in nitrogen concentration enhanced the (002) orientation. At pure N_2_, the films are fully (002) oriented. 

The final point is as follows. Our group varied the N_2_ concentrations and observed different results for two substrates. The 3C-SiC-on-Si (100) and 3C-SiC-on-Si (111) have 28.6% and 1% lattice mismatches with AlN, respectively. The former requires a lower N_2_ concentration of about 40% [8]. The latter produced consistent (002) film at all N_2_ concentration [69]. Our hypothesis is that the smaller lattice mismatch plays a significant role in invalidating the effect of N_2_ concentrations. 

### 5.5. Substrate Temperature 

Substrate temperature largely influences the kinetic energy available to the adatoms on the surface of the deposited films. This energy increases proportionally with temperature, which helps in depositing the highly c-axis oriented films. However, after the optimal point, a further increase in the substrate temperature increases the thermal stresses in the film because of the CTE difference when the film is cooled down to room temperature. In addition, there are many impurities that are absorbed through the surface of the film at high temperature. Both factors decrease the film’s crystal quality [73]. Table 2 shows the ranges of the substrate temperature. It starts from the room temperature and could go up to 1000 °C [74]. 

The effect of substrate temperature on AlN orientation has been reported by numerous groups. A few are highlighted herein. Yang et al. [25] deposited AlN on top of Molybdenum (Mo). They carried out the sputtering at the following substrate temperatures: 20, 200, 400, and 600 °C. As the deposition temperature increased from 20 to 400 °C, the crystal orientation changed from (101) to (002). When the temperature was further increased to 600 °C, the intensity of the AlN (0002) peak decreased. Their results point to the existence of optimum temperature ranges for AlN (002) film. This was supported by another group. Jin et al. [31] deposited AlN film on top of Si (100) substrate. They used substrate temperatures of 60, 160, 250, 340, 430, and 520 °C. The deposition rate increased from 60 °C to 250 °C and saturated afterwards at 21.78 nm/min. The XRD results showed that the highest peak of the (002) orientation was observed at 430 °C. The intensity of the AlN (002) peak decreased afterwards. Our own work found similar optimum temperature range between 350 to 400 °C [73]. We deposited AlN film on top of 3C-SiC-0n-Si (111) substrate.

Medjani et al. [55] investigated the combined effects of substrate temperature and substrate bias. They RF sputtered AlN films on top of Si (100) substrate by varying the temperature from 25 to 800 °C and bias voltage from 0 to −100 V. It was found that the lower substrate temperature and moderate bias voltage helps in the formation of the (002) plane. A bias voltage smaller than −75 V and a temperature of 400 °C resulted in the growth of the (100) plane. Another group studied the effect of substrate temperature and N_2_ concentration on the surface roughness of the AlN film. Guo et al. [75] observed that the roughness increased linearly with substrate temperature and N_2_ concentrations. 

There were not many works who performed the post annealing treatment on their sputtered films. Kar et al. [59] RF sputtered AlN film on top of Si substrate at 200 °C. After the samples cooled down, they subjected them to the annealing in N_2_ ambient for 90 s. The annealing temperature was increased from 400 to 1000 °C in steps of 200 °C. They observed that the intensity of AlN (002) diffraction peak increased until 800 °C, and then marginally decreased at 1000 °C. They also reported a small shift in the XRD diffraction peaks at higher annealing temperatures due to the residual stress. Also, the surface roughness (in rms) increased from 2.1 to 3.68 nm at annealing temperature of 400 to 1000 °C, respectively. Similar trend was found by Phan and Chung [34]. They annealed their samples for 1 h under N_2_ ambient at atmospheric pressure. They claimed superior performances of their Surface Acoustic Waves (SAW) devices after annealing. 

### 5.6. Film Thickness

This is a very interesting parameter. It is a general knowledge that the thicker the film, the less sensitive it is to the lattice mismatch between the AlN and its substrate. The most prominent work on this parameter is perhaps from Iriarte et al. [6], who systematically studied the effect of AlN film thickness on a wide variety of substrates. Figure 5 of their paper shows the trend. The crystal quality increased with increasing thickness. After 1 μm film thickness however, the FWHM of the rocking curve values of the deposited films remained stagnant. 

Other groups agree with that observation. Molleja et al. [26] investigated the role of AlN film thickness on its crystal quality and residual stress. The FWHM of the (002) diffraction peak decreased from 0.8 to 0.19 when the thickness was increased from 80 nm to 1.5 μm. They further reported that the thinner film had compressive stresses, while the thicker ones had tensile stress. Madrid et al. [30] supported Molleja’s group result by using the FWHM of the rocking curve measurement. 

Other groups extended this observation to the piezoelectric properties of their films. Ababneh et al. [41] studied the effect of film thickness on the values of the d_33_ and d_31_ piezoelectric coefficients. They noticed that by increasing the AlN film thickness from 600 nm to 2,400 nm, the values for the d_33_ and d_31_ increases from 3.0 and −1.0 to 5.0 and −1.8 pm/V, respectively. Martin et al. [76] supported this trend. They deposited AlN films on top of Pt electrode with two different thicknesses i.e., 35 nm and 2 µm. They reported the FWHM of the rocking curve decreased from 2.60 to 1.14° and the value of the d_33_ increased from 2.75 to 5.15 pm/V. It is worth noting that the same group wrote the highly cited article that measure the piezoelectric coefficient of AlN [77], and on the regrowth of AlN film [78]. 

### 5.7. Target to Substrate Distance 

The target to substrate distance could play a role in obtaining the c-axis AlN film. The exact degree of its significance has not been properly investigated. Most researchers agree that in the case of a smaller distance between the substrate and the target, the Al and N ions land on the substrate with higher kinetic energies and adatom mobilities due to the fewer collisions with other particles. On the other hand, when the distance is larger, these ions have multiple collisions with other particles before reaching the substrate. Thus, their kinetic energies and the subsequent adatoms mobilities decrease. From Table 2, target to substrate distances from 5 to 25 cm were used. The smaller distance is normally used for systems with higher powers. We further observed that most groups used the target to substrate distance from 3 to 8 cm for the sputtering power of 300 to 1000 W. 

We highlight several groups who investigated the influence of the distance, in combination with other sputtering parameters. Xu et al. [79] deposited AlN film on Si (111) substrates using DC sputtering. They studied the effects of the sputtering pressure, sputtering power, and target to the substrate distance on the crystal orientation of the AlN films. The distance between the substrate to target was varied from 3 to 12 cm. They reported that a low sputtering pressure and shorter distance helped to form the (002) plane. On the contrary, a higher sputtering pressure and longer distance are beneficial for the growth of the (100) plane. The findings from this group was supported by Chen et al. [80], as well as Cheng et al. [63].

### 5.8. Substrate Bias Voltage

In some sputtering experiments, the substrates were biased with an RF source to attract the Al+ ions towards them. Table 2 listed these works [19,27,55,62]. It is generally known that the amplitude of the bias voltage corresponds to the landing kinetic energies of these ions, which in turn determine the crystal orientation. The substrate bias could also be used to increase the deposition rate and to tune the residual stress of the AlN film. 

The formation of AlN (002) film requires higher biasing voltage when the low substrate temperature is used. Chu et al. [81] RF sputtered AlN thin films on top of glass substrates at room temperature. They applied different negative bias voltage from 0 to −320 V. The XRD diffraction showed a (002) oriented AlN up to −210 V. Afterwards, small diffraction peaks of AlN (002) and (100) planes were observed at a bias voltage of −240 V. Furthermore, the (002) plane vanished at the bias voltage of −320 V. The opposite trend is observed when the higher substrate temperature is used. One group reported a much lower amplitude to bias their substrate at the temperature of 200 °C. Iborra et al. [82] only needed a bias voltage of −24 V to achieve purely (002) oriented films. Finally, it is worth mentioning that Abdallah et al. [83] discusses the effect of compressive stress and ion bombardment on the orientation of the AlN film. 

### 5.9. Base Pressure

Al has high affinity for oxygen (O_2_), and the combination of both elements degrades the crystalline quality of the AlN (002) film [6]. Therefore, the chamber is kept in an almost-vacuum condition to minimize O_2_. This is accomplished by constantly pumping out the gas/vapor without pumping in the Ar or N_2_ gasses, a method that is commonly known as outgassing. Table 2 listed the values of the base pressure that were used by many researchers. The range is between 3.6 × 10^−3^ to 3.6 × 10^−7^ Torr for the successful deposition of AlN film. 

Cherng et al. [47] specifically studied the effect of outgassing on the deposition of AlN using pulsed-DC reactive sputtering of highly (002) oriented AlN thin film. They pumped down the system into the base pressure of 3 × 10^−6^ Torr, 1 × 10^−6^ Torr and 4 × 10^−7^ Torr before admitting the gas mixture into the chamber. They observed that both the FWHM of the rocking curve values and the residual stress became insensitive to the changes in the substrate temperature and deposition pressure at a lower base pressure. This was attributed to the less O_2_ contamination in the chamber.

### 5.10. Unbalanced Magnetron Configuration

Most published articles employ the conventional (balanced) magnetron configuration. In 1996, Window and Savvides [84] introduced the unbalanced configuration for sputtering system. It is able to increase the ion bombardments towards the substrate, at the price of rising substrate temperature. There are several groups who employed this technique for the deposition of AlN film. We highlight two of them in this review. Duquenne et al. [85] grew the film on top of Si (100) substrates with balanced and unbalanced configurations. By holding other parameters constant, the balanced system produced poly-crystal AlN with (100), (101) and (002) configurations. The unbalanced system produced only (002) plane, regardless of the N_2_ concentrations. This is attributed to the higher ion energy between 20–30 eV that is produced by that system. Another group compare the structural, morphological, and electric properties of AlN film that was grown on glass substrates in balanced and unbalanced configurations. Ke et al. [86] found that the latter configuration improves the AlN growth along the c-axis. The grains are denser and larger, and the film has smaller surface roughness, larger dielectric constant, and smaller leakage current. 

### 5.11. Sputtered AlN (002) films for difficult environments

We discussed piezoelectric energy harvesters in Section 1. They could be very useful to power up electronics systems for difficult environments where conventional supplies are not practical, and the amplitude of the vibrations generate sufficient energies. Difficult environments include extreme temperatures and pressures, high mechanical shock and highly corrosive mediums. AlN is a good material candidate due to its higher mechanical, thermal, and chemical stability i.e., corrosion resistance. 

There are many articles that characterized sputtered AlN’s mechanical and corrosion properties. Few examples are provided herein. Jian and Juang [87] employed nano-indentation technique to find out the hardness and deformation behavior of their sputtered c-axis film on sapphire substrate. They measured the value of film’s hardness and Young’s modulus as 16.2 and 243.5 GPa, respectively. Another group used AlN film as a protection material. Subramanian et al. [88] sputtered their AlN film as a coating material for mild steel (MS). That resulted in better wear resistance and a lower friction coefficient. We close this review paper by mentioning the newest applications of AlN film in life sciences. Reader is referred to a focus review on this topic in reference [89]. Towards that goal, one group RF sputtered AlN film and characterized its performance as biosensors, include its corrosion rate under saline solutions and compatibility with biological cells [90]. 

## 6. Conclusions

We analyzed around 80 journal articles that performed the reactive sputtering of c-axis AlN film on a wide variety of substrates and equipment. The data from Table 2 compiles the values of the sputtering parameters. These recipes could serve as reference points for any new work on the deposition of AlN film. Readers should be able to find the closest matches from Table 2 in term of their substrates and sputtering processes. In addition, the roles and ranges of the sputtering parameters in depositing c-axis oriented AlN film have been discussed. 

The flow chart in Figure 5 illustrates the roles of major sputtering parameters in depositing c-axis AlN film. This chart is self-explanatory, but two points should be highlighted. The values of the landing kinetic energies of the ions and the surface mobility of the adatoms determine the specific crystal orientations of the film. We would like the adatoms to arrange themselves in the (002) plane. In order to achieve that, we need to adjust the values of all sputtering parameters to achieve the right balance. The number of runs to get the best quality films should be reduced if readers study Section 5.1, Section 5.2, Section 5.3, Section 5.4, Section 5.5, Section 5.6, Section 5.7, Section 5.8, Section 5.9 and Section 5.10 well. 

## Figures and Tables

**Table 1:** Bulk AlN properties. The data are taken from references [13,14].

**Structural Properties**	Density (g/cm^3^)	3.257
	Elastic modulus (GPa)	330
	Elastic constant C_11_ (GPa)	410 ± 10
	Elastic constant C_12_ (GPa)	149 ± 1
	Elastic constant C_13_ (GPa)	99 ± 4
	Poisson’s ratio	0.22
	Crystal structure	Wurtzite
	Lattice constant (Å)	*a* = 3.112 *c* = 4.982
	Hardness (Kg/mm^2^)	1100
	Water absorption	None
**Optical Properties**	Density of states in conduction band (cm^−3^)	4.1 × 10^18^
	Effective hole mass	m*_hz_* = 3.53 m_0_ m*_hx_* = 10.42 m_0_
	Density of states in valence band (cm^−3^)	4.8 × 10^20^
	Optical phonon energy (meV)	113
	Refractive index (visible to IR)	~2.15
**Electrical Properties**	Breakdown field (V/cm)	1.2–1.8 × 10^6^
	Mobility of electrons/holes (cm^2^/V·s)	135/14
	Dielectric constant (static/high frequency)	8.5–9.14/4.6–4.84
	Energy band gap (eV)	6.13–6.23
	Resistivity (Ohm·cm)	10^15^
**Thermal Properties**	Thermal conductivity (W/m·°K)	140–180
	Coefficient of thermal expansion (×10^−6^/°C)	4.2–5.3
	Debye temperature (°K)	1150
	Melting Point (°C)	2200
**Piezoelectric Properties**	Piezoelectric coefficient e_15_ (C/m^2^)	−0.33~−0.48
	Piezoelectric coefficient e_31_ (C/m^2^)	−0.38~−0.82
	Piezoelectric coefficient e_33_ (C/m^2^)	1.26–2.1
	Relative permittivity coefficient ɛ_11_	9
	Relative permittivity coefficient ε_22_	9
	Relative permittivity coefficient ε_33_	11

**Table 2:** Summary of published works on the deposition of c-axis AlN films.

Authors [ref] (Year of Publication)	Substrate	Sputtering Type	Power (W)/Power Density (W/cm^2^)	Substrate Temperature (°C)	Sputtering Pressure (mTorr)	Base Pressure (mbar)	Nitrogen (%)	Total Gas (sccm)	Distance Target to Substrate (cm)	FWHM (°)	Deposition Rate (nm/min)	Film Thickness (µm)	Surface Roughness (nm)	Notes
Ohtsuka et al. [17] (2016)	sapphire	Pulsed DC magnetron sputtering	800/9.86	550	3 to 11	-	50	-	6	3.3 (rocking curve)	60	1.5	-	Effect of sputtering pressure on crystalline quality and residual stress.
Stan et al. [18] (2015)	Si	RF magnetron sputtering	**-**	50	1.5	-	25	40	3.5	11, 7.1 (rocking curve)	19	0.6, 1.1	0.5 to 1.6	Investigated electric and pyroelectric properties of deposited films.
Wang et al. [19] (2016)	Glass	DC magnetron sputtering	170/9.55	400	9	-	15	35	4.7	-	66.5	8.35	48	Effects of substrate temperature and bias voltage on crystal orientation.
Jiao et al. [20] (2015)	Si (100), Si (111), SiO_2_, and amorphous Si (α-Si)	RF Magnetron sputtering	150, 200, 250, 300/5.3, 7.07, 8.84, 10.6	25	5	5 × 10^−4^	50, 66, 75, 80	60	6	-	-	-	4.22	Effect of various Si substrates on film quality. Effect of RF power and gas flow on residual stress and film quality.
Bi et al. [21] (2014)	Si (100)	DC magnetron sputtering	460/5.34	400	3	1 × 10^−10^	85	22.8	7.5	1.63 (rocking curve)	7.5	1.8	-	Measured the longitudinal piezoelectric coefficient of deposited films.
Shih et al. [22] (2014)	Si_3_N_4_/Si	RF Magnetron sputtering	200, 250, 300/NA	300	5, 10, 15	6.6 × 10^−8^	60		5	-	11.2	1.9	6.42	Effect of RF power and sputtering pressure on film quality. Fabricated SAW device.
Stoeckel et al. [23] (2014)	Si (100)	Pulsed DC magnetron sputtering	865/7.6	350	5.25	-	80		7.5	0.39 (diffraction peak)	0.204	-	-	Measured transverse piezoelectric coefficient d_31_ using laser Doppler vibrometer (LDV).
Lim et al. [24] (2001)	Si, Ru/Si and ZnO/Si	RF magnetron sputtering	-	150	0.5	-	50	-	5	5.96, 4.05, 1.19 (rocking curve)	8.4	0.5 to 0.6	-	Efects of Si, Ru/Si and ZnO/Si substrates on the crystal quality of AlN film.
Yang et al. [25] (2014)	Mo/Si (100)	RF magnetron sputtering	200/7.07	20 to 600	7.5	2 × 10^−7^	50		6.5	2.4 (rocking curve)	-	-	-	Effect of substrate temperature on film quality.
García Molleja et al. [26] (2013)	SiO_2_/Si (100)	DC reactive magnetron sputtering	100/11.68	25	3	2 × 10^−8^	30		3	0.8 to 0.19 (diffraction peak)	-	1.5	-	Effect of film thickness on residual stress and film quality.
Monteagudo-Lerma et al. [27] (2013)	C-sapphire	RF reactive sputtering	100–175/5.1–8.94	400	3.5	1 × 10^−5^	100		10.5	1.63 (rocking curve)	-	-	0.4	Effect of substrate bias, RF power and substrate temperature on deposited films.
Aissa et al. [28] (2013)	Si (100)	DC Magnetron sputtering	150/7.66	Room temp	3	6 × 10^−5^	35	40	3	-	20 to 40	580 for DCM and 980 for HiPMS	-	Comparison of the structural properties and residual stress as a function of sputtering pressure deposited via DCM and HiPMS.
Kale et al. [29] (2012)	Si, copper, quartz	RF magnetron sputtering	100/1.27	200	6	1 × 10^−7^	50	-	5	-	-	-	-	Structural and electrical properties as a function of N_2_ concentration.
Rodríguez-Madrid et al. [30] (2012)	Microcrystalline diamond	Balanced magnetron sputter deposition	700/NA	25	3	6.6 × 10^−7^	75	12	4.5	2 (rocking curve)	-	3	4.2	Effect of film thickness on film quality for SAW devices.
Jin et al. [31] (2013)	Si (100)	DC magnetron sputtering	270/9.55	430	3	5 × 10^−6^	50	100	-	2.259 (rocking curve)	21.78	1	1.97	Effect of substrate temperature on structural properties.
Ababneh et al. [32] (2012)	Ti/Si0_2_/Si	DC magnetron sputtering	1000/3.183	-	-	4 × 10^−3^	100	-	6.5	0.3 (diffraction peak)	-	0.6	1	Investigate the effect of the thickness and surface roughness of the Ti substrate to the crystal quality of the AlN film.
García-Gancedo et al. [33] (2011)	IR/Si (100)	Pulse DC magnetron sputtering	1200/6.79	400	1.2	2.3 × 10^−5^	70	-	-	1.8 (rocking curve)	40	1.5	7	Sputtered AlN film to make bulk acoustic wave (BAW) sensors for biometric applications.
Phan and Chung [34] (2011)	Si (100)	Pulse DC magnetron sputtering	-	25	3.5	5 × 10^−7^	90	-	8	0.21 (diffraction peak)	8	-	-	Effect of post annealing treatment for acoustic wave applications.
Singh et al. [35] (2011)	N-type Si (100)	RF magnetron sputtering	100,200,300/2.19,4.38,6.57	25	5, 10, 20	2 × 10^−6^	50	-	5	-	-	-	-	Effect of sputtering pressure on deposited films.
Cardenas-Valencia et al. [36] (2011)	Sapphire	Pulse DC magnetron sputtering	205/8.2	860	1.25, 1.5	-	50	11.5	-	0.32 (diffraction peak)	200	-	-	Novel sputtering method as the magnet was embedded in the target.
Iriarte et al. [37] (2011)	Au/Si substrate	Pulsed DC reactive ion beam	900/NA	50	2	6.6 × 10^−8^	55	65	5.5	1.3 (rocking curve)	-	-	1.43	AlN growth on top of Au buffer layer.
Moreira et al. [38] (2011)	P-Si (100)	DC magnetron sputtering	50/2.04	50	3	2 × 10^−8^	27	80	-	-	70	-	-	Electrical characterization of AlN prepared at different N_2_ concentration.
Singh et al. [39] (2011)	Glass, Si, oxidized Si, Al–SiO2–Si, Cr– SiO2–Si, and Au–Cr–SiO2–Si	RF magnetron sputtering	100,200,300/2.19,4.38,6.57	25	5,10,20	2 × 10^−6^	100	-	-	0.32–0.40 (diffraction peak)	-	1	7.7	Comparison of AlN sputtered at different power and pressure on various substrates.
Subramanian et al. [40] (2011)	Si (100), glass	DC magnetron sputtering	180/NA	200	1.5	1 × 10^−6^	50	-	6	-	-	-	-	Mechanical and optical properties of deposited films.
Ababneh et al. [41] (2010)	Si (100)	DC Magnetron sputtering	300, 500/1.59, 3.18	150–200	1.5, 4.5	5 × 10^−6^	-	50	6.5	0.29–0.35 (diffraction peak)	6–12	0.5	-	Effect of N_2_, sputtering pressure and DC power on deposited films.
Taurino et al. [42] (2017)	SiO_2_/Si (100)	RF magnetron sputtering	150/NA	-	3 to 18	2 × 10^−7^	60	-	8	-	-	0.2 and 0.5	-	Control the deposition pressure to switch from (101) to (002) planes.
Vashai et al. [43] (2009)	Silicon	Pulse DC magnetron sputtering	1500/3.18–11.45	300	2.1	-	100	50	6	1.2–2.4 (rocking curve)	-	0.28 pa	-	Influence of sputtering parameters on film quality.
Clement et al. [44] (2009)	Iridium layers	Pulse DC magnetron sputtering	10000/NA	400	5	6.6 × 10^−8^	80	-	5	2 (rocking curve)	24	-	-	Comparison of BAW resonator performance on Mo and Ir substrates.
Cherng et al. [45] (2008)	Si (100)	Pulse DC magnetron sputtering	1500/NA	-	-	4 × 10^−6^	40–100	-	7	2 (rocking curve)	-	-	-	Two step deposition method by varying power, pressure and N_2_ concentration.
Abdallah et al. [46] (2008)	Si (100)	DC reactive magnetron sputtering	-	25	3	1.3 × 10^−5^	30	-	3	0.14–0.4 (FWHM of diffraction peak)	40	-	-	Effect of thickness on film quality.
Cherng and Chang [47] (2008)		Pulse DC magnetron sputtering	600/NA	25	2	5.3 × 10^−7^	60	-	7	2 (rocking curve)	-	1.6	-	Role of base pressure in AlN deposition.
Chiu et al. [48] (2007)		DC reactive magnetron sputtering	1000–1600/5.42–8.77	250–450	3–7.5	-	30–100	-	2–12	2.7° (rocking curve)	12	2	1	Effect of substrate temperature, sputtering power and N_2_ concentration on AlN films.
Kano et al. [49] (2006)	Si, SiO_2_	RF magnetron sputtering	460/NA	100	3.75	-	50	-	-	8.3 (rocking curve)	-	-	-	Measured piezoelectric coefficient.
Venkataraj et al. [50] (2006)		DC reactive magnetron sputtering	500/11.2	Room temp	6	1.3 × 10^−4^	variable	-	5.5	0.4 (diffraction peak)	60	-	-	Effect of N_2_ concentration on structural, optical and mechanical properties of deposited films.
Benetti et al. [51] (2006)	Diamond	RF magnetron sputtering	500/2.74	200–500	3	-	100	-	5	0.4 (diffraction peak)	-	-	-	Effect of sputtering temperature.
Kar et al. [52] (2006)	Si (100)	RF magnetron reactive sputtering	400/NA	200	4.5	3 × 10^−6^	variable	-	5	-	5.5	-	2.4	Effect of nitrogen concentration of film quality.
Umeda et al. [53] (2006)	Si (100)	RF magnetron sputtering	1300–1800/7.38–10.2	200	1.5	1 × 10^−6^	70	60	5	1.4 and 2.1 (rocking curve)	-	-	1.7	Effect of sputtering parameters on residual stress
Guo et al. [54] (2006)	Sapphire	RF magnetron sputtering	100–250/1.27–3.18	100	5	1 × 10^−7^	40	9	-	-	8	-	6	Effect of sputtering power.
Medjani et al. [55] (2006)	Si (100)	RF magnetron sputtering	150/NA	25, 400,800	3.75	4 × 10^−9^	14	18	6.5	-	-	-	-	Effect of substrate temperature and bias voltage on the crystallite orientation.
Vergara et al. [56] (2006)	Si (100)	RF magnetron sputtering	-	900–1300	6.75	2.5 × 10^−7^	50	-	-	-	-	-	-	Effect of rapid thermal annealing on piezoelectric response.
Kar et al. [57] (2006)	P-type Si (100)	RF magnetron sputtering	400/NA	100–400	4.5	3 × 10^−6^	80	-	8	-	-	-	2	Role of sputtering temperature.
Jang et al. [58] (2006)	P-type Si	RF magnetron sputtering	100/1.23	300	2–5.25	6.6 × 10^−5^	-	-	-	-	-	-	-	Effect of rapid thermal annealing in oxygen ambient.
Kar et al. [59] (2005)	Silicon, copper, quartz	RF magnetron reactive sputtering	400/NA	200	4.5	3 × 10^−6^	80	-	5	0.25 (diffraction peak)	-	-	2.1–3.68	Influence of rapid thermal annealing on morphological and electrical properties.
Iriarte et al. [60] (2005)	Al, Mo, Ti, TiN, and Ni	Pulse DC magnetron sputtering	900/4.97	-	2	6.6 × 10^−8^	70	-	5.5	1.3 (rocking curve)	-	-	-	Comparison of metallic substrates on crystal orientation.
Zhang et al. [61] (2005)	Si (100), Si 111)	RF magnetron sputtering	200–500/1.76–4.42	350	6	3.7 × 10^−7^	100	-	8	-	-	-	-	Effect of sputtering power on crystal quality and strain in film.
Sanz-Hervas et al. [62] (2005)	Al, Si0_2_, Cr, Mo and Ti	RF reactive sputtering	800/NA	-	7	-	50	-	-	-	-	-	-	Effect of substrate bias on crystal quality.
